# Medical Information Protection in Internet Hospital Apps in China: Scale Development and Content Analysis

**DOI:** 10.2196/55061

**Published:** 2024-06-21

**Authors:** Jiayi Jiang, Zexing Zheng

**Affiliations:** 1 Law School Central South University Changsha China

**Keywords:** hospital apps, privacy policy, personal information protection, policy evaluation, content analysis

## Abstract

**Background:**

Hospital apps are increasingly being adopted in many countries, especially since the start of the COVID-19 pandemic. Web-based hospitals can provide valuable medical services and enhanced accessibility. However, increasing concerns about personal information (PI) and strict legal compliance requirements necessitate privacy assessments for these platforms. Guided by the theory of contextual integrity, this study investigates the regulatory compliance of privacy policies for internet hospital apps in the mainland of China.

**Objective:**

In this paper, we aim to evaluate the regulatory compliance of privacy policies of internet hospital apps in the mainland of China and offer recommendations for improvement.

**Methods:**

We obtained 59 internet hospital apps on November 7, 2023, and reviewed 52 privacy policies available between November 8 and 23, 2023. We developed a 3-level indicator scale based on the information processing activities, as stipulated in relevant regulations. The scale comprised 7 level-1 indicators, 26 level-2 indicators, and 70 level-3 indicators.

**Results:**

The mean compliance score of the 52 assessed apps was 73/100 (SD 22.4%), revealing a varied spectrum of compliance. Sensitive PI protection compliance (mean 73.9%, SD 24.2%) lagged behind general PI protection (mean 90.4%, SD 14.7%), with only 12 apps requiring separate consent for processing sensitive PI (mean 73.9%, SD 24.2%). Although most apps (n=41, 79%) committed to supervising subcontractors, only a quarter (n=13, 25%) required users’ explicit consent for subcontracting activities. Concerning PI storage security (mean 71.2%, SD 29.3%) and incident management (mean 71.8%, SD 36.6%), half of the assessed apps (n=27, 52%) committed to bear corresponding legal responsibility, whereas fewer than half (n=24, 46%) specified the security level obtained. Most privacy policies stated the PI retention period (n=40, 77%) and instances of PI deletion or anonymization (n=41, 79%), but fewer (n=20, 38.5%) committed to prompt third-party PI deletion. Most apps delineated various individual rights, but only a fraction addressed the rights to obtain copies (n=22, 42%) or to refuse advertisement based on automated decision-making (n=13, 25%). Significant deficiencies remained in regular compliance audits (mean 11.5%, SD 37.8%), impact assessments (mean 13.5%, SD 15.2%), and PI officer disclosure (mean 48.1%, SD 49.3%).

**Conclusions:**

Our analysis revealed both strengths and significant shortcomings in the compliance of internet hospital apps’ privacy policies with relevant regulations. As China continues to implement internet hospital apps, it should ensure the informed consent of users for PI processing activities, enhance compliance levels of relevant privacy policies, and fortify PI protection enforcement across the information processing stages.

## Introduction

### Background

The emergence and rapid expansion of hospital apps represents a significant milestone in the evolution of global health care services [1,2], especially during the COVID-19 pandemic [3-7]. These digital platforms provide a range of medical services, from digital consultations [8,9] to telemedicine [10] and digital care management [6,11]. Their growing use reflects a trend toward digital health solutions as enhanced, accessible, and cost-efficient health care services [12].

However, the rise of hospital apps has been accompanied by substantial concerns regarding patient privacy and data security [13-15], as with other mobile health (mHealth) applications [16,17]. The apps’ extensive collection of personal health and medical information, as well as the sensitive nature of that data, suggest a need for comprehensive, rigorously enforced regulations to prevent unauthorized access, misuse, and disclosure. In regions like the United States [18,19] and the European Union, similar digital health initiatives have been developed that focus on interoperability, patient-centricity, and adherence to strict data protection regulations, such as the HIPAA (Health Insurance Portability and Accountability Act) in the United States [20,21] and the General Data Protection Regulation in the EU [22,23]. Striking a balance between leveraging the benefits of digital health services and ensuring the confidentiality and integrity of patient information remains an ongoing challenge in the industry.

In China, the response to the evolution of digital health care has been swift, aided by the prevalence of mobile internet [24] and the development of mHealth services [25-27], as evidenced by the 2014 launch of the country’s first officially approved internet hospital in Guangdong province [28,29]. This milestone, coupled with the enactment of several “internet plus healthcare” policies [30], has led to a surge in digital hospital apps, bringing the terms “internet diagnosis” and “internet hospitals” into the national health care context [31-33]. “Internet diagnosis” encompasses medical services provided in digital form by registered doctors, including consultations for certain common and chronic diseases and “internet plus” family doctor services [34]. Hospital apps are divided into two categories: (1) digital extensions of traditional hospitals and (2) stand-alone entities operated by internet enterprises [34]. The former involves local doctors and patients, whereas the latter combines the resources of various medical institutions to expand service to patients across different locations.

Internet hospital apps offer digital consultation, appointment scheduling, diagnosis and treatment of common and chronic diseases, and medical guidance, as well as prescription and delivery of medications and other treatments [32,35]. These apps have significantly enhanced health care by addressing the disparities in resource distribution and access across the mainland of China’s large population [26,31,36] and improved overall patient experiences by enhancing communication, transparency, and efficiency [37]. The COVID-19 pandemic further underscored the efficacy of digital health care providers, which facilitated crucial health care services for prevention and control in the pandemic’s early stages [38-42].

However, the existing application of these apps presents significant challenges to patient information protection [43,44]. First, sensitive personal information (PI) generated during medical visits, such as biometric and health data, is vulnerable to unauthorized sharing and cyberattacks, which can lead to privacy breaches [45,46]. Second, the complexity of integrating and applying health data weakens individuals’ control over their health information once it transforms into big health care data [47-49]. Finally, the difficulty of implementing and upholding informed consent is compounded by the lack of unified industry standards and the realities of “algorithmic black boxes,” which often leave individual patients in a relatively disadvantaged position [50,51].

To manage these issues, China has established a regulatory framework to protect PI. Since 2017, the Information Security Technology-Personal Information Specification (PI Specification) has been adopted as a voluntary standard for PI protection practice by all kinds of enterprises in information processing activities [52-54]. In addition, the Personal Information Protection Law (PIPL), guided by the Chinese Civil Code [55] and effective starting November 11, 2021, serves as the nation’s first comprehensive national PI legislation. The PIPL specifies the rights of individuals and the obligations of PI processors [56,57]. The Chinese government has also made a specific commitment to protect personal health information and prohibits illegal processing, trade, or disclosure of personal health information in article 92 of the Law on the Promotion of Basic Medical Care, Hygiene, and Health, enacted on June 1, 2020.

Internet hospital apps represent a critical intersection of PI and digital technology, which underscores the urgent need for scrutiny of these providers’ privacy policies within a framework that balances self-regulation and governmental oversight [58,59]. Privacy policies delineate how PI processors collect, use, disclose, and manage a customer or client’s data [60]. They are also the primary grounds for the transparent data processing requirements mandated by privacy-related regulations [61]. Drawing inspiration from contextual integrity (CI) theory, we also investigated how the privacy policies articulated and adhered to the norms of information flow [62]. In keeping with Nissenbaum’s [63] assertion that privacy “is preserved when informational norms are respected and violated when informational norms are breached,” we set the basis of evaluation with a focus on the norms and values that govern appropriate flows of PI.

Previous research on privacy compliance of mHealth apps in different countries has identified gaps between rules for privacy protection and the apps’ implementations in various aspects, such as lack of complete privacy policies, lack of informed consent, and insufficient protection of sensitive data [64-73]. Such investigations have also raised concerns about internet hospital apps’ uneven design quality and the challenges in minimizing users’ cognitive load while ensuring information security [74,75]. However, these studies have not thoroughly examined web-based hospitals’ compliance with China’s comprehensive legal framework for PI protection.

This study uses a legal framework to assess the compliance of internet hospital apps’ privacy policies with China’s PI-related regulations. The Methods section elaborates on the collection and selection of sample apps, describes the development of an evaluation scale based on relevant policy documents, and outlines the procedures for app assessment and scoring. In the Results section, we present the compliance scores of sample apps. The Discussion section contextualizes these results within the broader landscape of mHealth app privacy compliance, underscoring the importance of legal compliance in the evolving digital health landscape.

### Objective

In this study, we aimed to (1) collect the privacy policies of internet hospital apps developed for users in the mainland of China, (2) develop a scale based on the provisions stipulated in the PIPL, PI Specification, and rules of the hospitals, (3) assess the compliance of the privacy policies within the regulatory framework of PI protection, and (4) offer recommendations for improving the legal compliance of internet hospital apps’ privacy policies to enhance PI protection in the evolving landscape of mHealth innovation. This study contributes to the global discussion on balanced policies for PI protection in digital health initiatives in the postpandemic era and provides insights for policymakers, hospital app providers, and users across different countries while highlighting the importance of improving legal compliance and strengthening enforcement.

## Methods

### Study Design

We conducted a content analysis of the privacy policies of internet hospital apps available in the Apple App Store in the mainland of China and evaluated their compliance with the PIPL, PI Specification, and hospital app rules. Drawing from CI theory, we considered the adherence of internet hospital apps’ privacy policies to PI norms as essential to PI protection.

### App Selection and Inclusion Criteria

In this study, we focused on the privacy policies of internet hospital apps available in the Apple App Store and tailored for the market of Chinese mainland. To identify relevant apps, we used the keyword “internet hospital” (*hu lian wang yi yuan* in Chinese) to search on Diandian (*Dian Shu Ju* in Chinese), a prominent mobile data analytics platform in China. We conducted the search on November 7, 2023.

**Figure 1:**
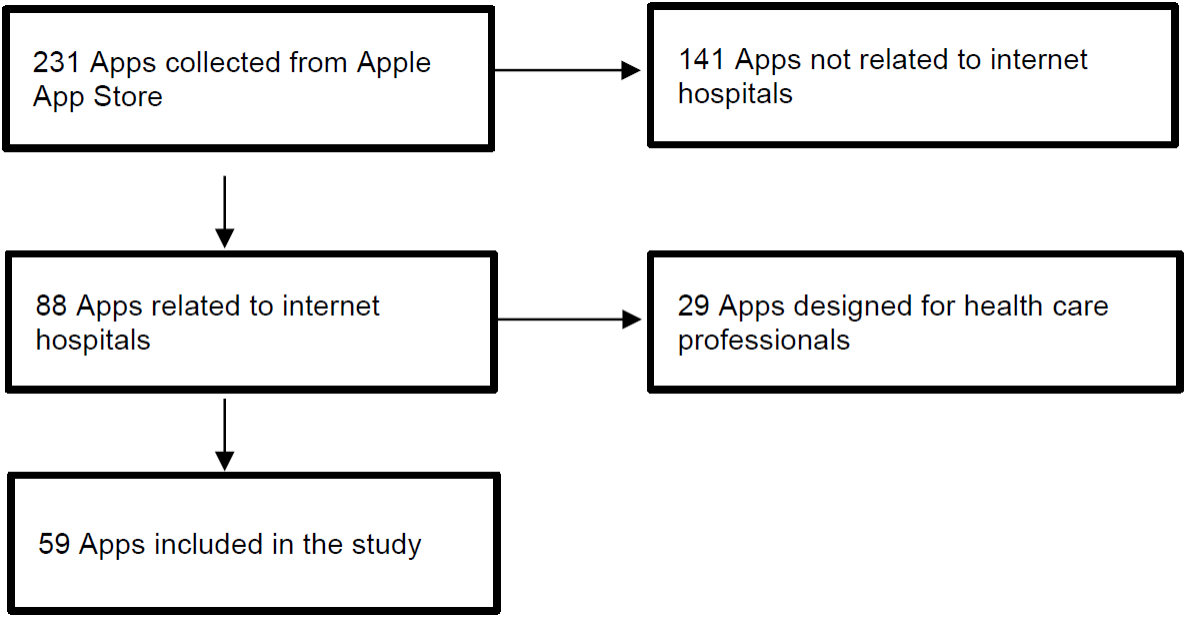
Filtering procedure for the selection and inclusion of hospital apps.

The apps included in the sample fell under the following definitions: (1) apps or platforms specifically developed to provide a range of hospital app services, and (2) apps intended for use by the general population rather than health care professionals. Excluded apps fit the following: (1) apps designed for health care professionals managing internal hospital operations, and (2) apps with scope or functionality unrelated to hospital app services, such as those dedicated to health insurance, maintaining a healthy lifestyle, or health education and popular science. The initial search resulted in a total of 231 apps, out of which 59 met the inclusion criteria and were included in the final analysis upon review (Figure 1). We obtained and reviewed the full text of corresponding privacy policies as text files or screenshots from the sample apps between November 8 and 23, 2023.

### Development of the Compliance Evaluation Scale

#### Overview

We systematically developed a compliance evaluation scale to assess the privacy policies of internet hospital apps against the PIPL, PI Specification (GB/*t* 35273-2020), and the Rules for Regulation of Internet Diagnosis and Treatment Management (for Trial Implementation). This process entailed the following sections.

#### Comprehensive Review

First, we obtained and meticulously reviewed the full text of the PIPL, PI Specification, and the aforementioned rules to understand the comprehensive regulatory framework governing PI protection in hospital apps.

#### Indicator Development

Based on the information processing activities delineated in these policy documents, we identified level-1 evaluation indicators encompassing critical processing stages such as PI collection and usage; PI storage and protection; PI sharing, transfer, disclosure, and transmission; PI deletion; individual rights; and PI processor duties. In addition, we introduced “general attributes” as an additional level-1 indicator to evaluate the overall transparency and ongoing maintenance efforts.

#### Indicator Elaboration

We then translated the specific chapters and clauses of these policy documents into a more granular set of 26 level-2 indicators and 70 level-3 indicators, which provided a detailed framework for our evaluation. Each level-2 indicator represented a crucial component within the respective PI processing stage—our level-1 indicators. For example, in the stage of PI collection and usage, we followed different rules for general and sensitive PI as stipulated in the PIPL, and further developed 2 level-2 indicators to evaluate the collection and usage of general PI and sensitive PI. Moreover, we established level-3 indicators to assess the specific compliance requirements as detailed under each level-2 indicator. For example, concerning the collection and usage of sensitive PI, we identified 7 level-3 indicators according to the PIPL, which included: highlighting sensitive PI, stating the specific purpose, clarifying the sufficient necessity, implementing stringent protective measures, communicating the implications of processing sensitive PI, obtaining separate explicit consent for processing sensitive PI, and requiring explicit consent for processing PI of minors.

#### Operational Definitions and Examples

To ensure clarity and consistency in our assessment and support the application of our evaluation criteria, we have included brief explanations, example sentences, and references to the relevant provisions of the policy documents for all the level-3 indicators in Multimedia Appendix 1.

### Scoring and Evaluation Procedure

We adopted a binary scoring system for level-3 indicators, awarding a score of 1 for privacy policies that adequately addressed a given indicator and 0 for those that did not. This allowed us to calculate the compliance rate for each level-3 indicator based on the proportion of policies scoring “1” from our app sample. We then calculated the scoring rate for each level-2 indicator as the arithmetic mean of the scoring rates for its associated level-3 indicators. Similarly, we determined the compliance rate for the level-1 indicators as the mean of the scoring rates of the corresponding level-2 indicators, which reflects the overall compliance of each app in specific stages of the information processing activities. The overall compliance of each app’s privacy policy was quantified by aggregating the scores of all level-3 indicators and converting this total into a percentage to denote the app’s compliance level.

To ensure the reliability of our evaluation, 2 independent raters (JJ and ZZ) were engaged to assess the privacy policies of all 59 internet hospital apps between November 8 and 24, 2023. To gauge interrater reliability, both raters independently evaluated a randomly selected subset of 20 apps (34% of the total), achieving an intraclass correlation coefficient of 0.986 (*P*<.001), indicating nearly perfect agreement. Following this assessment, the raters convened to discuss score discrepancies in their initial evaluations. After this, the raters divided the remainder of the apps equally and applied the unified standards to ensure scoring consistency.

## Results

### Sample Collection

We accessed 59 internet hospital apps available in the Apple App Store for Chinese mainland users by registering as users with our own identity documents and mobile phone numbers. We obtained the full text of 52 privacy policies. A small but significant percentage of apps (7/59, 12%) altogether lacked a separate privacy policy, a fundamental requirement for safeguarding PI. This absence is a critical oversight and represents direct noncompliance with established PI protection laws, suggesting an urgent need for these apps to develop and implement comprehensive privacy policies.

### Compliance Evaluation

The overall compliance landscape among the 52 assessed privacy policies was mixed. The mean compliance score of all policies was 73 of a possible 100 (SD 22.4%). Moreover, 36 apps (69%) surpassed the mean score, whereas 16 apps (31%) fell below.

**Figure 2:**
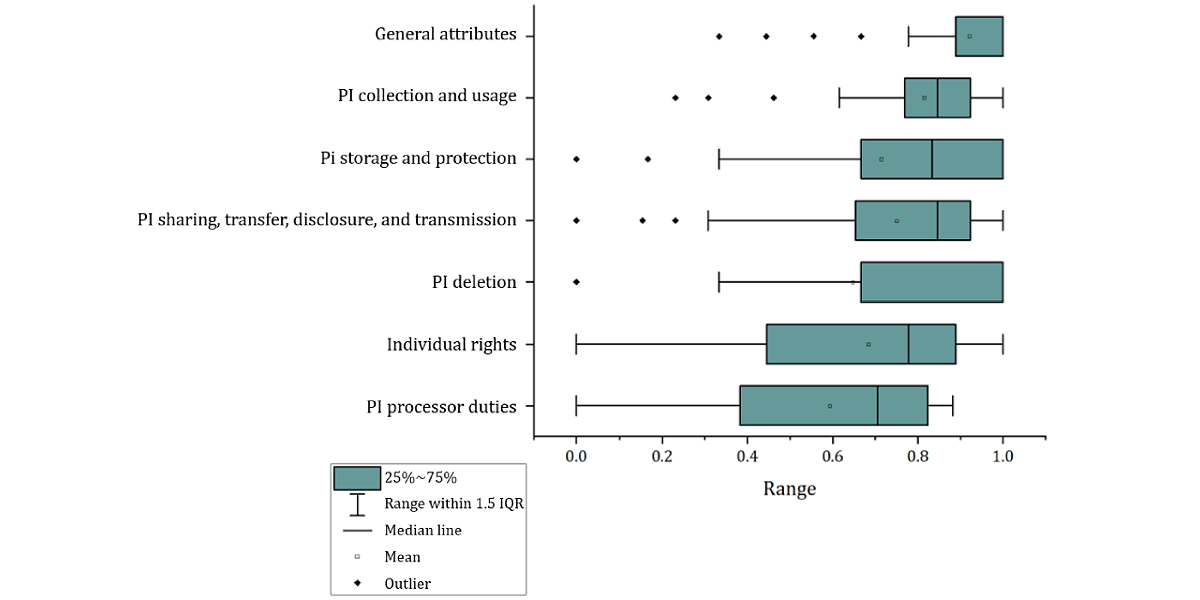
Compliance evaluation scores of internet hospital apps for level-1 indicators. PI: personal information.

The evaluation results for level-1 and level-2 indicators are listed in Figure 2 and Table 1. Level-1 indicators were ranked by score from highest to lowest, as follows: general attributes (mean 92.1%, SD 16.5%); PI collection and usage (mean 81.5%, SD 17.9%); PI sharing, transfer, disclosure, and transmission (mean 75%, SD 25.2%); PI storage and protection (mean 71.5%, SD 30.7%); individual rights (mean 68.4%, SD 31.5%); PI deletion (mean 64.7%, SD 34.8%); and PI processor duties (mean 59.4%, SD 28.4%). The names and evaluation results for each app are listed in Multimedia Appendix 2.

The privacy policies’ general attributes (mean 92.1%, SD 16.5%) scored high, indicating effective efforts in transparency and maintenance. For level-2 indicators, PI processors and services recorded an impressive compliance rate of 95.2% (SD 20.2%), indicating a majority of the privacy policies effectively identified the parties responsible for processing PI and providing services. Policy transparency was a standout area, with a perfect score of 100% (SD 0%) reflecting the apps’ commitment to clear and open communication with users. Policy maintenance also emerged as a strong suit, scoring 84.6% (SD 30.3%). This suggests a significant proportion of apps were proactive in updating their privacy policies, a vital aspect of best practices following the implementation of the PIPL. Specifically, 25 apps updated their privacy policies after the PIPL came into force. However, a concerning 12 apps failed to mention either the effective or updated date of their policies, whereas 15 updated their privacy policies before the PIPL came into effect.

Regulations for a description of the collection and usage of general PI had an average compliance rate of 90.4% (SD 14.7%). This indicates the majority of internet hospital apps were conscientious in describing how general PI is collected and used within their service functions. Our evaluation found all the reviewed privacy policies specified the purpose and methods of collecting and using PI, demonstrating a high level of transparency. Additionally, a substantial 90% (n=47) of apps provided a list of the types of PI collected, while 83% (n=43) of the policies clarified the consequences of not providing PI. In terms of differentiating between essential and nonessential PI for services, compliance stood at 69% (n=36). Although significant clarity was currently provided, an opportunity still remained for apps to enhance user understanding of the purpose and optional nature of PI collection.

Meanwhile, the scoring rate of collection and usage of sensitive PI was lower (mean 73.9%, SD 24.2%). We observed strong compliance rates for describing specific purposes (n=48, 92%), protective measures (n=46, 88.5%), implications (n=43, 83%), and necessity (n=41, 79%) of processing sensitive PI. Most assessed apps required explicit consent for processing minors’ PI (n=43, 83%). However, the requirement to obtain separate explicit consent for processing sensitive PI revealed a significant gap, with only 23% (n=12) of apps complying.

In the PI storage and protection stage (mean 71.5%, SD 30.7), the scoring of level-2 indicators varied slightly. The compliance rate of storage security was 71.2% (SD 29.3%). Most apps explained potential security risks (n=46, 88.5%) and organizational management measures. Fewer than half (n=24, 46%) outlined the compulsory level of technical security measures. As for security incidents (mean 71.8%, SD 36.6%), although a significant portion of apps committed to notifying users (n=43, 83%) and reporting security incidents (n=42, 81%), just over half of PI processors (n=27, 52%) committed to assuming legal responsibility in the event of such an incident.

In the stage of PI sharing, transfer, disclosure, and transmission (mean 75%, SD 25.2%), the scoring rate of level-2 indicators varied substantially. For public disclosure (mean 93.3%, SD 24.1%), we observed high compliance in specifying conditions for potential public PI disclosure (n=49, 94%) and requiring separate consent for such practices (n=48, 92%). These rates indicated a high degree of transparency and respect for user consent for public disclosure. As for the compliance rate of PI sharing and transfer (mean 77.5%, SD 30%), most privacy policies introduced information about PI recipients (n=37, 71%), the types of PI transferred (n=38, 73%), and the safety precautions adopted in advance (n=37, 71%). In addition, most apps explained the purposes (n=44, 85%) and methods (n=44, 85%) of PI transfer, described the rules governing PI transfer during specific events (n=37, 71%), and required separate consent for sharing or transferring PI (n=44, 85%). Regarding cross-border transmission (mean 71.2%, SD 43.1%), most apps specified PI storage locations (n=39, 75%), whereas fewer mentioned compliance with relevant cross-border transmission laws (n=35, 67%). However, the compliance rate of subcontracting PI processing was low (mean 51.9%, SD 27.7%). Although most apps committed to supervising the subcontracted PI processing activities (n=41, 79%), only a quarter (n=13, 25%) required separate consent for these activities.

**Table 1:** Compliance evaluation scores of internet hospital apps for level-2 indicators.

Evaluation results on level-2 indicators	Compliance rate (%), mean (SD)
PI^a^ processors and service	95.2 (20)
Policy transparency	100 (0)
Policy maintenance	84.6 (30)
Collection and use of general PI in service functions	90.4 (15)
Collection and use of sensitive PI in service functions	73.9 (24)
Storage security	71.2 (29)
Security incidents	71.8 (37)
Subcontracting of PI processing	51.9 (28)
PI sharing and transfer	77.5 (30)
Public disclosure	93.3 (24)
Cross-border transmission	71.2 (43)
Retention period	76.9 (42)
Deletion and cessation	58.7 (38)
Inquiry of PI	80.8 (39)
Obtain copies of PI	42.3 (49)
Correction of PI	80.8 (39)
Deletion of PI	80.8 (39)
Explanation regarding PI processing	82.7 (38)
Consent withdrawal	51.9 (34)
Deregistration	76.9 (42)
Consent exception scenarios	67.3 (46)
PI protection officer disclosure	48.1 (49)
Compliance audits	11.5 (38)
Impact assessment procedures	13.5 (15)
Request management	72.9 (36)
Complaint management	64.7 (40)

^a^PI: personal information.

In the stage of PI deletion (mean 64.7%, SD 34.8%), most privacy policies stated the PI retention period (mean 76.9%, SD 42.1%). In contrast, the scoring rate for deletion and cessation was lower (mean 58.7%, SD 37.6%). Although most apps committed to PI deletion or anonymization after a retention period (n=41, 79%), only 20 apps (38.5%) required third parties to delete PI or cease processing after the same period.

Concerning individual rights (mean 68.4%, SD 31.5%), most apps explained individuals’ various rights effectively, including the rights to inquire about (n=42, 81%), correct (n=42, 81%), and delete PI (n=42, 81%); cancel the account (n=40, 77%); withdraw or modify consent (n=41, 79%); and request an explanation of the privacy policy (n=43, 83%). However, only 22 apps recognized the right of users to obtain copies of their PI (n=22, 42%) and only 13 explained the right to refuse business marketing using automated decision-making. A majority of apps (n=35, 67%) listed exceptions for obtaining consent as provided by applicable laws or administrative regulations.

Concerning PI processor duties, we found a compliance rate of 59.3% (SD 28.4%). Fewer than half of the apps appointed a PI officer and disclosed their information in their privacy policies (n=25, 48%). A quarter of the apps (n=13, 25%) presented impact assessment procedures, whereas 11.5% of apps (n=6) engaged in compliance audits. Many apps provided methods for individuals to inquire about (n=41, 79%), correct (n=41, 79%), and delete PI (n=41, 79%); clarify PI processing rules (n=43, 83%); cancel their account (n=41, 79%); withdraw or modify consent (n=40, 79%); and understand limits on the use of automated decision-making (n=29, 56%). However, fewer than half of all the studied apps provided methods to obtain copies of PI (n=21, 40%). Although many apps provided a means for lodging complaints by disclosing contact information (n=37, 71%), fewer committed to addressing these complaints within the stated time limits or explained the methods of dispute resolution (n=32, 61.5%).

## Discussion

### Principal Findings

We developed the evaluation scale to align with the characteristics of internet hospital apps, drawing from the essential CI parameters of context, attributes, actors, and transmission principles. We set the context in the realms of internet diagnosis and hospitals and categorized the attributes of PI, emphasizing the distinction between sensitive and general PI processing activities. Actors encompassed both app users and PI processors, including third-party entities outlined in the privacy policies. We translated the transmission principles of lawfulness, legitimacy, necessity, good faith, minimal impact, openness, and transparency into indicators that aligned with PI-related regulations.

Our review of 52 privacy policies from internet hospital apps in the mainland of China reveals a varied spectrum of compliance. The compliance score of the apps’ privacy policies varied (mean 73%, SD 22.4%), with some apps demonstrating robust compliance, whereas others fell short. This suggests a need for enhanced regulatory oversight and standardized practices. We also identified variations in legal compliance across different stages of the information processing activities, as shown in Figure 2 and Table 1. This underscores the varying application of PI-related regulations in digital hospital apps, raising concerns about users’ potential exposure to privacy risks.

First, our analysis indicates a notable gap between compliance rates for sensitive PI protection (mean 73.9%, SD 24.2%) and general PI protection (mean 90.4%, SD 14.7%), raising significant concerns regarding the provision of stringent safeguards for sensitive PI [76,77]. This gap is especially concerning given the PIPL (specifically section 2, chapter II) mandates special protection for sensitive PI. The inadequate compliance in this area also potentially diminishes users’ awareness and understanding of the risks associated with the processing of their sensitive PI. Article 28 of the PIPL stipulates that PI processors may only process sensitive PI with a specified purpose, sufficient necessity, and stringent protective measures. Alarmingly, the practice of seeking explicit consent for processing sensitive PI—a fundamental requirement for lawful processing and respecting user rights—is not as widespread as it should be, implying a pervasive reliance on blanket consent strategies among digital hospital apps. These findings also suggest privacy policies often fail to provide the necessary clarity for users to understand the distinctions between various types of PI and the specific reasons for their processing. Enhancing privacy policies to offer more detailed explanations would not only align with the PIPL’s mandate but also elevate the standard of user empowerment, enabling individuals to make informed decisions about their PI.

Second, the fact that many apps did not fully elucidate the role of third-party subcontractors or the conditions of PI sharing, transferring, or deletion in privacy policies may hinder users’ understanding of the destinations and protections of their PI, which could consequently affect their trust and the integrity of their informed consent [78]. The lack of detailed disclosure about PI processors (including involved third parties) and protocols for PI sharing and transfer, particularly in critical scenarios like mergers or acquisitions, underscores a disconnect between regulatory intentions and the operational realities of data governance within these digital platforms. In addition, the apps’ handling of PI deletion remains challenging and becomes more complex when third-party subcontracting activities are involved [79,80]. It is particularly problematic when privacy policies do not clearly communicate how these third parties are managed or if they are held to the same rigorous standards of PI protection as the primary PI processors.

Third, we found users’ rights to inquire about, correct, and delete personal data, along with other user-centric controls, were generally recognized within the apps’ privacy policies. The rights of individuals to manage their PI are paramount in the domain of digital health [81,82]. However, a deeper look into the specifics of these policies uncovers a gap in the acknowledgment of users’ right to obtain copies of their own PI, a provision stipulated in article 45 of the PIPL. More concerning is the fact that only a quarter of the apps addressed the right to refuse business marketing through automated decision-making, even though article 24 of the PIPL calls for transparency, fairness, and the right to receive an explanation and be able to opt out of such marketing. A lack of explanation of these crucial rights might inadvertently hinder app users from fully realizing their entitlements under PI protection norms.

Finally, our findings reveal inadequacies in how internet hospital apps execute PI-related responsibilities, even though the roles and responsibilities of PI processors are central to the protection of PI [83]. The absence of clear methods for users to obtain copies of their PI or comprehensive explanations of automated decision-making processes stands in stark contrast to the protective intent of the PIPL. Moreover, the relatively low scores of PI protection officer disclosure, compliance audits, and impact assessment procedures suggest a concerning lapse in institutional oversight. Such critical mechanisms are essential for the proactive identification of vulnerabilities and agile adaptation to emergent technological threats.

### Recommendations

Since the creation of China’s first internet hospital, the nation’s government has shown commendable support for the industry in its policy making [25,32,84,85]. China’s “internet plus” policy paves the way for a promising future for internet hospital apps beyond their role in the prevention and control of the COVID-19 pandemic [41,86]. Constructing health and medical big data requires the aggregation and integration of personal health care information, so it is essential to address PI risks posed by big data technology. The public-interest nature of health and medical information in areas like infectious disease control, medical research, and public safety further underscores the importance of the reasonable use and sufficient protection of PI [87,88]. However, the rapidly growing sector of internet diagnosis and hospitals still grapples with gaps in patient information protection [43,89], necessitating a balanced approach that judiciously considers both the advantages of processing PI and the inherent challenges associated with PI protection [90].

There is a pressing need to standardize obtaining informed consent in internet hospital services. The prevalent absence of explicit consent, particularly in subcontracting processes, raises significant privacy concerns, ranging from unauthorized data collection to inadequate user disclosure and excessive data harvesting [91-94]. Individuals often find their control over their own health information reduced, especially as it becomes integrated into big data [48,95]. Drawing on CI’s focus on the principles of actors and transmission, it is essential to adopt a dynamic consent model to reinforce granular control over PI. Implementing robust privacy impact assessments and creating transparent platforms for sharing privacy policies can further enhance public trust [50].

Improving the compliance of privacy policies and their enforcement mechanisms requires adherence to CI principles across the information processing stages. This includes ensuring clarity in the collection and use of PI, enhancing protection for PI storage, and promoting transparency in the sharing, transfer, and deletion of PI. Emphasizing the internet diagnosis and hospitals’ context and the attributes of PI can help in distinguishing between sensitive and general PI, ensuring PI processing activities are aligned with users’ needs and rights. Expanding acknowledgment of individual rights concerning users’ PI, a core aspect of CI, involves not only recognizing users’ rights to access, correct, and delete their PI, but also ensuring they are adequately informed about the purposes and methods of PI processing. This can be achieved through regular audits, impact assessments, and the appointment of PI protection officers, which ensure internet hospital apps not only comply with legal standards but also respect ethical digital health practices.

### Limitations

This study, while comprehensive in scope, encountered several limitations common in privacy policy analyses. First, our methodology primarily relied on content analysis of privacy policies, which may not fully capture the actual practices and implementation effectiveness of these policies. There is often a gap between what is stated in policy documents and how those policies are executed. Thus, the findings may not accurately reflect the on-the-ground application of apps’ privacy standards. Second, this study was confined to the examination of publicly available privacy policies, without delving into the apps’ technical backend and data-handling processes. This limitation means we could not assess the real-world effectiveness of the stated privacy measures or the security of the apps’ data management systems. Future researchers could benefit from incorporating technical audits, user-experience studies, and automated analysis, which could provide a more holistic and dynamic view of privacy protection in internet hospital apps.

### Conclusions

Our comprehensive evaluation of privacy policies from 52 internet hospital apps in the mainland of China highlights a landscape marked by varied compliance with relevant regulations. Despite some apps demonstrating adherence to legal standards, notable gaps persist, especially in protecting sensitive PI, obtaining informed consent, and clearly delineating individual rights. Inspired by CI theory, in this study, we underscore the urgent need for enhanced regulatory oversight, standardized privacy practices, and a commitment to user empowerment through transparent, comprehensive privacy policies. Addressing these challenges is critical, not only for protecting PI but also for fostering trust and facilitating the sustainable growth of digital health care services in China and other countries.

## Appendix

Multimedia Appendix 1 This chart provides the 3-level indicator scale developed to evaluate the compliance status of the sample apps.

Multimedia Appendix 2 This chart provides a list of 59 internet hospital apps that met the inclusion criteria.

## Abbreviations

CI: contextual integrity
HIPAA: Health Insurance Portability and Accountability Act
mHealth: mobile health
PI: personal information
PIPL: Personal Information Protection Law
